# Genome Sequences of *Pseudomonas amygdali* pv. tabaci Strain ATCC 11528 and pv. lachrymans Strain 98A-744

**DOI:** 10.1128/genomeA.00683-15

**Published:** 2015-06-18

**Authors:** Haeyoung Jeong, Joseph W. Kloepper, Choong-Min Ryu

**Affiliations:** a Super-Bacteria Research Center, Korea Research Institute of Bioscience and Biotechnology (KRIBB), Daejeon, Republic of Korea; b Biosystems and Bioengineering Program, University of Science and Technology (UST), Daejeon, Republic of Korea; c Department of Entomology and Plant Pathology, Auburn University, Auburn, Alabama, USA

## Abstract

Certain pathovars of *Pseudomonas amygdali*, which is newly reclassified from *Pseudomonas syringae* by DNA-DNA hybridization and ribotyping, cause many serious diseases of major crop plants. Herein, we present draft genome sequences of *P. amygdali* pv. tabaci strain ATCC 11528 and *P. amygdali* pv. lachrymans strain 98A-744.

## GENOME ANNOUNCEMENT

Most bacterial strains classified under the species *Pseudomonas syringae* are plant pathogens that grow epiphytically on plant hosts. Earlier studies gave rise to the idea of the “*Pseudomonas syringae* complex,” a single species with many populations capable of infecting specific hosts (1). However, multilocus sequence analyses suggest there are multiple species and pathovars of *P. syringae* (2). Specifically, DNA relatedness and ribotyping studies replaced *P. syringae* pathovars tabaci, lachrymans, phaseolicola, mori, and others into *P. amygdali* (3). Moreover, an alternative allocation method that relies on concatenated *gyrB* and *rpoD* genes also exists (4). To understand the genomic basis underlying host-pathogen interaction and to help genome information-based taxonomy for the *P. amygdali* group, pathovars tabaci (strain ATCC 11528) and lachrymans (strain 98A-744) were chosen for genome sequencing.

*P. amygdali* pv. tabaci ATCC 11528 was purchased from the American Type Culture Collection (Manassas, Virginia, USA). *P. amygdali* pv. lachrymans 98A-744, originally obtained from the Department of Horticulture, University of Wisconsin, Madison, USA (5), had been used for laboratory uses. The genomes were sequenced at the National Instrumentation Center for Environmental Management from Seoul National University (Seoul, Republic of Korea) using the Illumina HiSeq 2000 platform. One hundred one nucleotide (nt) paired-end reads, approximately >500× coverage, were produced from ca. 460 bp-long genomic libraries. *De novo* assembly was performed with the A5-miseq pipeline (6). The initial assemblies resulted in 18 scaffolds (total length 6,129,081 bp, *N*_50_ 542,503 bp) for ATCC 11528, and 193 scaffolds (total length, 6,175,433 bp; *N*_50_, 85,995 bp) for 98A-744. To improve the latter assembly, a 3-kb jumping library was constructed with the 5500 SOLiD mate pair library kit and Ion Torrent PGM sequencing was performed, which was carried out by GenoTech Corporation (Daejeon, Republic of Korea). Reads generated from one PGM 314 chip were split into 470,694 paired reads (average read length, 78.6 bp) using the CLC Genomics Workbench 8.0 (CLC bio). A5-miseq assemblies and mate pair reads (quality trimming-passed 288,586 reads) were scaffolded with the SSPACE Premium v2.3 (BaseClear), followed by automatic gap filling using the GapFiller (7). The final assembly for 98A-744 consists of 64 scaffolds, with a total 6,276,659 bp and *N*_50_ of 346,697 bp. Although there are already two independent genome sequence assemblies for ATCC 11528, and another two assemblies for *P. amygdali* pv. lachrymans (strains M301315 and M302278), our assemblies are much closer to the complete level. Automatic genome annotation was carried out using the NCBI Prokaryotic Genome Annotation Pipeline (PGAP) service.

The two pathovars possess 146 genes for type III secretion system and effectors indicating that *P. amygdali* as well as *P. syringae* use the system to suppress pathogen-associated molecular pattern-elicited immunity (PTI) and effector-triggered immunity (ETI). Both pathovars include the biosynthesis gene cluster of coronatine that acts as a plant immune inhibitory toxin and stomata closure determinant (8, 9).

### **Nucleotide sequence accession number**s. 

These whole-genome shotgun projects of ATCC 11528 and 98A-744 have been deposited at DDBJ/EMBL/GenBank under the accession numbers LCWS00000000 and LCWT00000000, respectively. The versions described in this paper are versions LCWS01000000 and LCWT01000000.
